# Oxidized HDL and Isoprostane Exert a Potent Adipogenic Effect on Stem Cells: Where in the Lineage?

**DOI:** 10.16966/2472-6990.109

**Published:** 2016-04-27

**Authors:** Stephen J Peterson, Luca Vanella, Angelica Bialczak, Joseph Schragenheim, Ming Li, Lars Bellner, Joseph I Shapiro, Nader G Abraham

**Affiliations:** 1 Weill Cornell Medical College, Department of Medicine, New York Methodist Hospital, Brooklyn, NY 11215, USA; 2 Departments of Medicine & Pharmacology, New York Medical College, Valhalla, NY 10595, USA; 3 Marshall University, Joan C. Edwards School of Medicine, Huntington, WV 25701, USA

## Stem Cell Development

**Figure 1:**
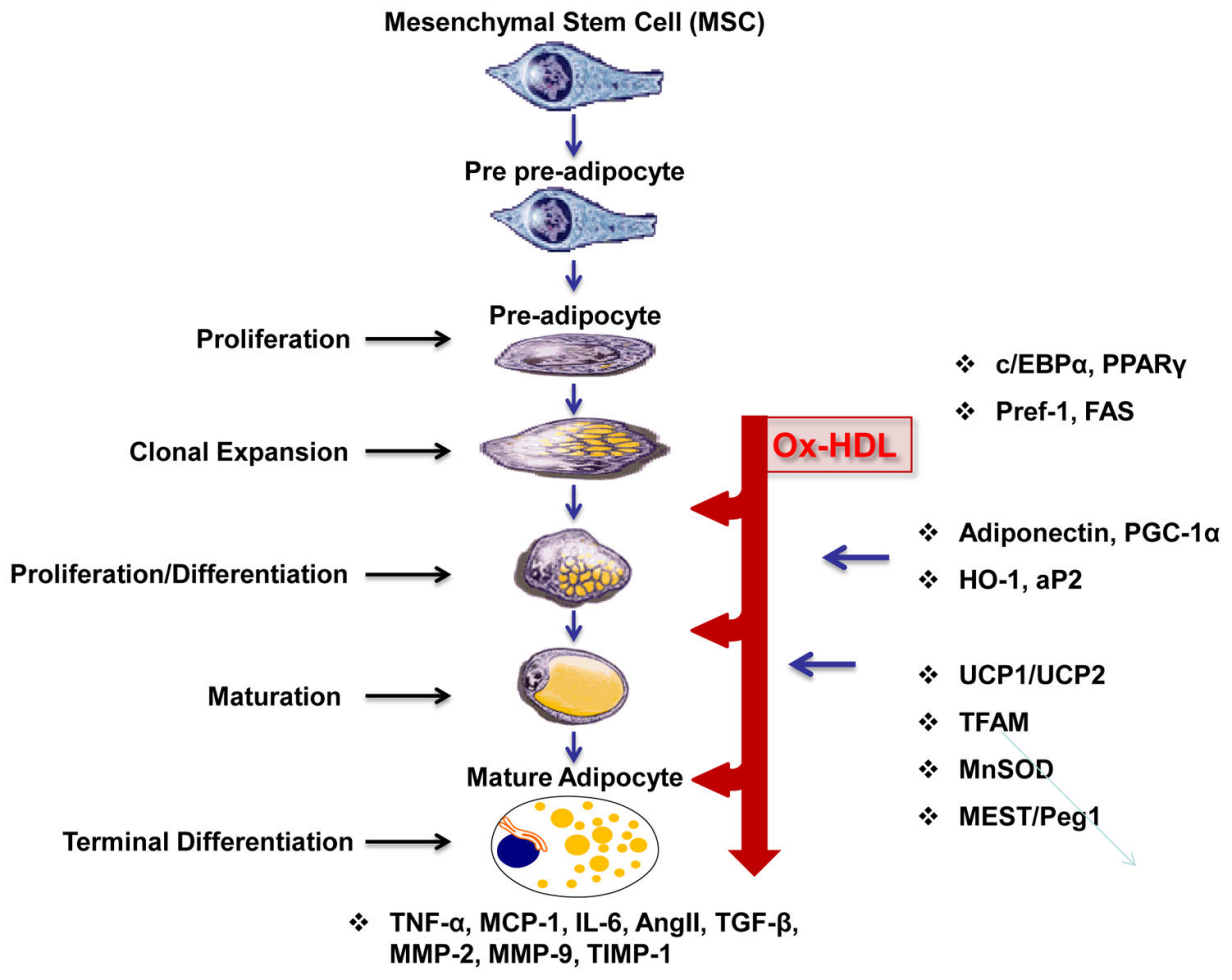
Schematic presentation of MSCs giving rise to adipocyte differentiation. MSCs can differentiate into adipocytes when placed in the adipogenesis medium *in vitro*. Various adipokines including Ang II, Leptin, TGFβ, VEGF, FGF, HGF, TNF, Adiponectin, MMP-2, MMP-9 and IGF-1 are secreted from adipocytes. Particular molecular events accompanying each stage of differentiation are indicated to the right, with the imprecise interval in each stage reflected as indicated.

The development of adipocytes in mice and humans follows a well-defined pathway that commences with a common pluripotent mesenchymal stem cell (MSC), ie., adipogenesis [1]. The early steps of the pathway leading to the generation and the commitment of MSCs to an adipocyte lineage are unknown. Hypothetically, the determination of the fate of MSCs occurs early in cell differentiation (“commitment”) and involves the interplay of intrinsic (genetic) and environmental (local and systemic) conditions that ultimately define the fate of the cell. Factors that determine MSC proliferation and differentiation also govern early adipocyte development and function. Currently, little is known about this process; from MSC-to-preadipocyte differentiation. However, the steps governing the transition from preadipocyte to adipocyte differentiation are not well defined (Figure 1). During adipogenesis MSCs or preadipocytes differentiate into lipid-laden adipocytes [2]. Ox-HDL increases adipogenic properties with a marked effect on the last step of adipocyte-terminal differentiation and release of adipokines including 20-HETE and Ang II.

MSCs were initially identified in postnatal human bone marrow and have been used to model differentiating mesoderm. It is believed that the MSCs give rise to a common early precursor (pre-adipocyte, Adipoblast), which, in turn, develops into the committed white and brown preadipocyte that under appropriate stimulatory conditions, differentiate into mature adipocytes of different types [3]. The transition from preadipocyte to adipocyte involves four stages: growth arrest, clonal expansion, early differentiation and terminal differentiation [4]. Adipocytes regulate glucose homeostasis [5] and adipocyte dysfunction results in the secretion of decreased levels of adiponectin and decreased glucose uptake, leading to insulin resistance [6].

## Obesity and Ox-HDL

Obesity is also linked to the metabolic syndrome, which is associated with a dyslipidemic profile that includes hypertriglyceridemia and low plasma high-density lipoprotein cholesterol (HDL-C). Accumulated evidence suggests that HDL enhancement plays a beneficial role in maintaining glucose homeostasis via insulin dependent and independent pathways. Low Density Lipoprotein Cholesterol (LDL-C) and HDL-C levels have become the accepted biomarkers in the evaluation of the risk of CVD, CAD, and even CKD [7,8]. Recent studies have suggested that HDL function is more important than total levels of HDL and that remodeling and dysfunction likely contribute to increased risk of CVD, CKD, and CRS.

High fat diets increase LDL and glucose levels [9] which are both reversed by an increased expression of the antioxidant gene, heme oxygenase (HO-1). In another model of high fat (HF) diets in hypertensive rats, LDL is increased and this is prevented by induction of HO-1 by a number of cobalt compounds including cobalt protoporphyrin [10]. Similar observations are described for male and female mice [11]. These observations are attributed to increases in ROS in adipose tissue and liver that may involve increases in Ang II and 20-HETE, which are major sources of ROS [12]. Deletion of angiotensinogen in hepatocytes markedly decreased blood pressure [13]. Angiotensinogen has been synthesized by 3T3-F442A cells and hydrolyzed to ANG l and ANG II in adipocytes [14], and its deletion from adipose tissue resulted in a decrease in blood pressure elevation in obese mice [15]. In another study, increases in antioxidants decrease the Ang II-mediated increase in ROS [16–18]. These reports suggest that targeting the Ang II system may have therapeutic value. The increase in ROS is considered a contributing factor in Ox-LDL [19] in contrast to an increase of HO-1, which inhibits atherogenesis [20] and atherosclerotic lesion in LDL receptor (−/−) mice [21], reviewed in [22].

Dysfunctional HDL can result from both free radical attack and oxidation of ‘good’ HDL, leading to Ox-HDL (‘bad’ HDL) [23–25]. Lipids and lipoproteins are the primary targets of free radical damage [26], which results in lity and CVD and cardiac events.

## Process of MSCs differentiation to Adipocytes

### HO-1 effect Plasma LDL and HDL

**Figure 2:**
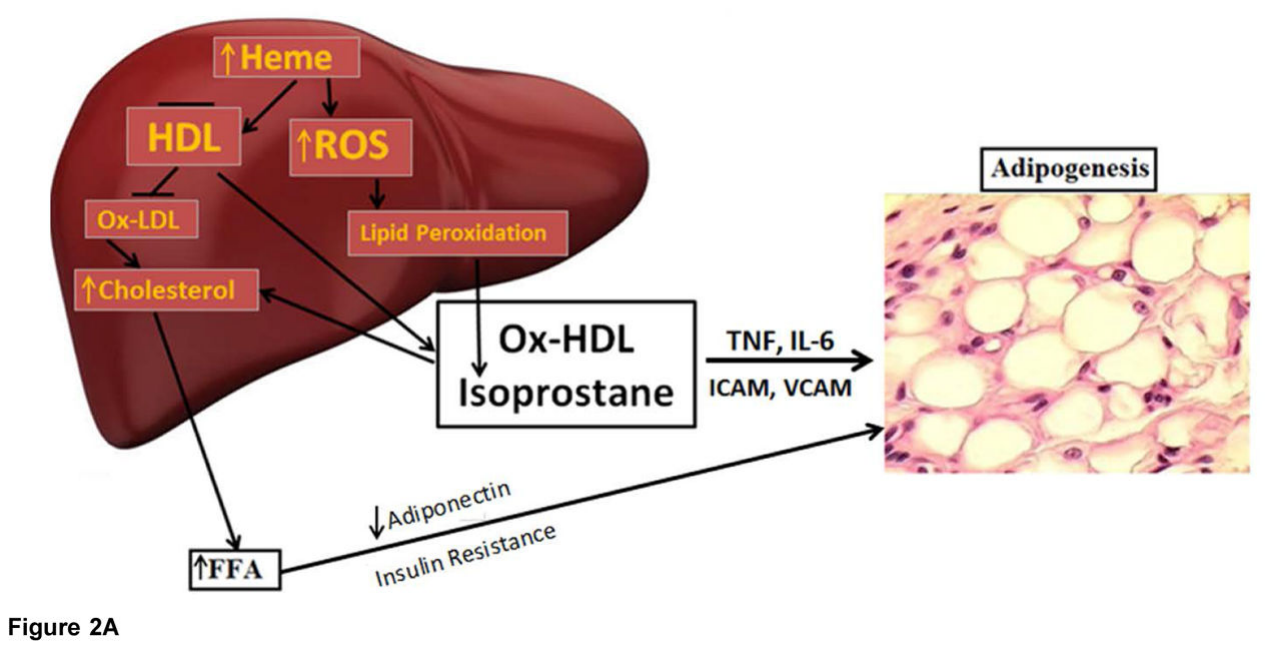

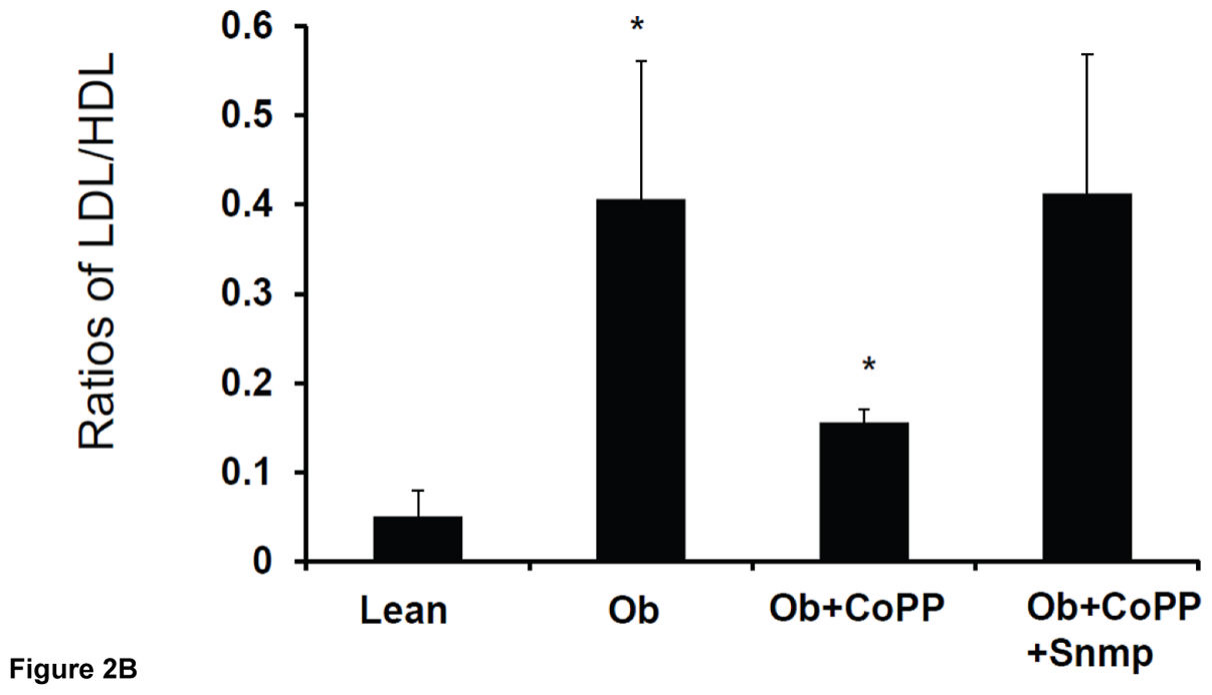
**Figure 2A**. HO-1 decrease ratios of LDL/HDL in obese mice treated with CoPP, obese mice display high levels of LDL, while treatment with CoPP, for 4 weeks decrease LDL, that is reversed by inhibition of antioxidant HO-1 **Figure 2B**. HO-1 decreases ratios of LDL/HDL in obese mice treated with CoPP, obese mice display high levels of LDL, while treatment with CoPP, for 4 weeks decreases LDL, that is reversed by inhibition of antioxidant HO-1.

We believe that levels of antioxidants will change the ratio of LDL and HDL in mice. As shown in figure 2A and 2B, the ratios of plasma LDL and HDL is significantly higher in obese mice than in lean mice (0.41 + 0.15 *vs* 0.05 + 0.02, *p<0.05). An increase of HO-1 and antioxidant properties [12,39] by CoPP decreased the ratio (0.15 + 0.01 *vs* 0.41 + 0.15, *p<0.05). Inhibition of HO-1 and increase of antioxidant by SnMP blocked the effect of CoPP on obese mice.

## The Effect of Ox-HDL and Isoprostanes on Adipogenesis

**Figure 3:**
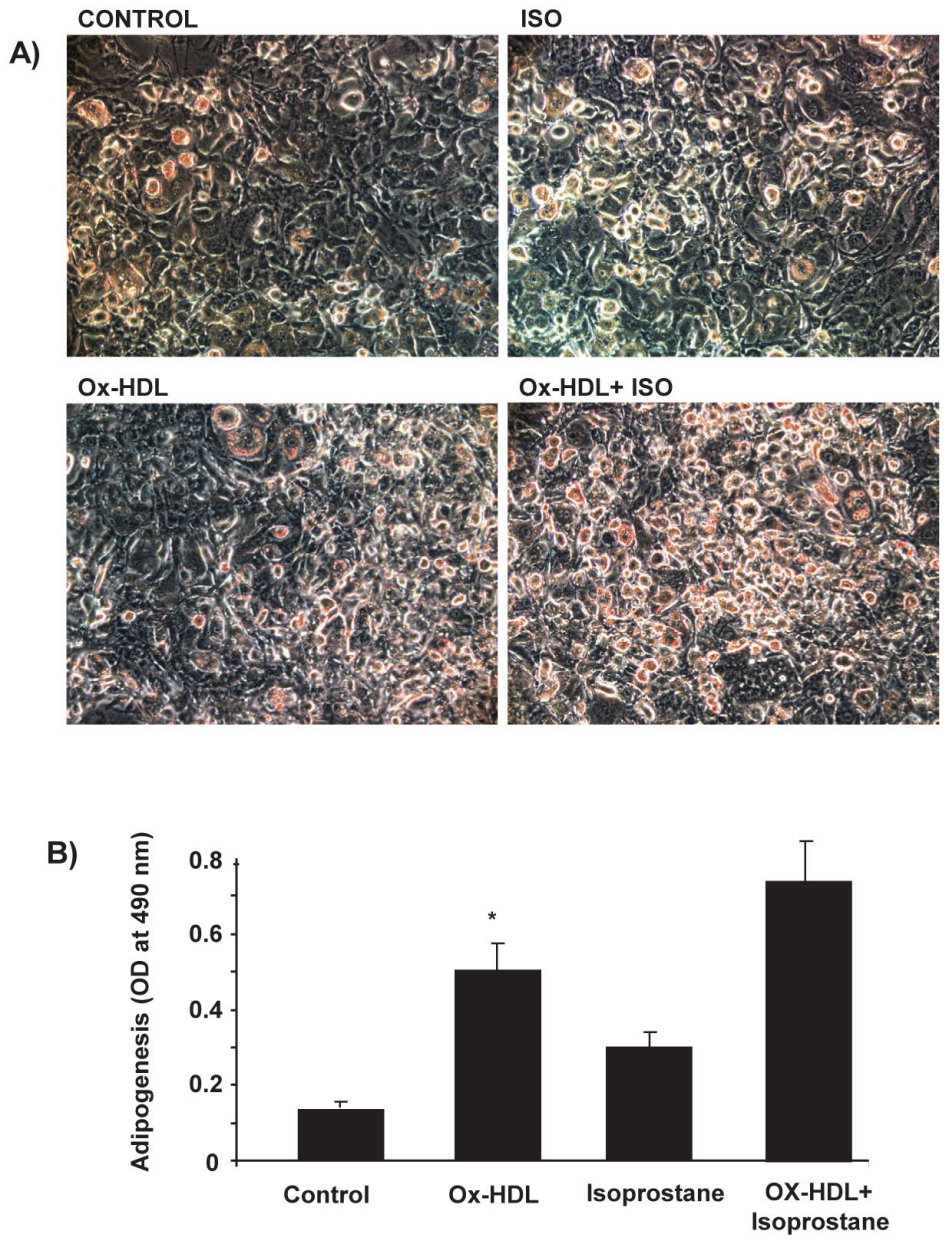
Adipogenic effect of oxidized HDL and isoprostane on MSC-derived adipocytes. Adipogenesis from human MSC was detected by Oil Red O staining and absorbance was measured as described [37,39]. *p<0.05 versus control.

We examined the levels of LDL to HDL in mice treated with CoPP, which increases HO-1-derived bilirubin levels. Since obesity is associated with a ISSN 2472-6990 decrease of antioxidants, we propose that this will result in an increase in levels of Ox-HDL as Ox-HDL is increased in cardiac events. We examined the effect of Ox-HDL and isoprostanes on adipogenesis in the human adipocyte by measuring Oil Red O stained lipid droplet area after 10 days of treatment (Figure 3). The level of Oil Red O stained lipid droplets increased after treatment with Ox-HDL, isoprostanes, and a combination of the two. Quantification of Oil Red O stained cells showed an increase in lipid droplets in the presence of both Ox-HDL and isoprostanes compared with control p<0.05 and Ox-HDL. This effect proved to be synergistic, p<0.05 (Figure 3). These results were confirmed in mice (results not shown).

**Figure 4:**
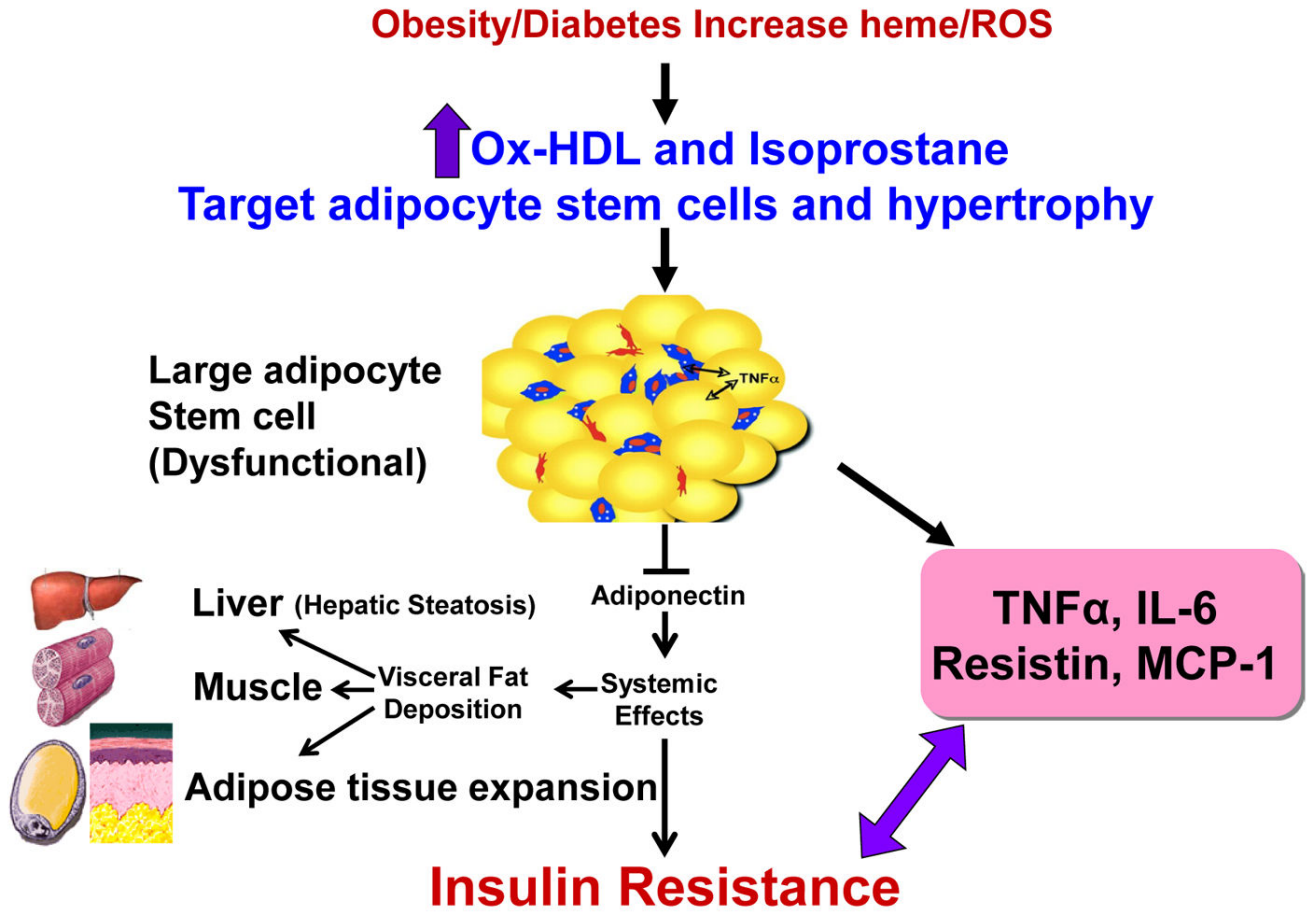
Schematic representing the increase in ROS by high fat, glucose or excessive heme levels that in turn increase the generation of oxidized HDL and isoprostane. Enlargement of adipocytes causes alterations in the secretion of adipokines. Increased adipocyte size can lead to deleterious alterations in insulin sensitivity caused by a decrease in adiponectin secretion and the induction of inflammatory mediators.

Figure 4 is a schematic that shows the release of the inflammatory cytokines IL-6, and TNF and ROS. ROS increases lipid peroxidation with increased levels of Ox-HDL, LDL and isoprostane. Excess heme, needed for adipocyte differentiation and terminal differentiation, also increases ROS. Hyperglycemia in the obese will also increase the levels of ROS (Reviewed in [12]). With the down regulation of HO-1 in obesity, heme catabolism is decreased. ROS targeting adipocyte stem cells and hypertrophy occurs in several animal models of obesity which leads to an increase of inflammatory adipokines, a decrease in adiponectin, liver and muscle fat deposit and insulin resistance.

This review demonstrates that Ox-HDL and isoprostane exert marked increases in adipogenesis in human adipocyte stem cells. Ox-HDL is associated with an increase in adipocyte expansion and adiposity and, as such, is a determinant of obesity and its related disorders. There are several ways in which Ox-HDL can be formed. One way is during the process of differentiating adipocytes. This process begins with a high food intake, early hyperglycemia occurs resulting in an increase in cellular heme due to a decrease in the levels of HO-1 (reviewed in [12]). Heme is a pro-oxidant and a source of ROS which contribute to an increase in NO uncoupling by iNOS induction. The induction of iNOS causes the formation of peroxynitrite which is responsible for lipid peroxidation and inhibition of protein and enzyme function and increased Ox-HDL levels. A prime example is a decrease in the levels of HO-1 which, in turn, decreases bilirubin levels. Bilirubin is a potent antioxidant and patients with elevated bilirubin levels display a lower risk of cardiovascular disease and have higher levels of HDL (reviewed in [31]).

There are a number of mechanisms by which obesity increases the levels of Ox-HDL. These occur during the process of differentiating adipocytes that requires glucose, which is a major source of ROS. Furthermore, myeloperoxidase is responsible for generating excessive levels of ROS [32] with a resultant increase in lipid peroxidation which converts LDL and HDL to oxidized products with an expansion of adipogenesis.

We and others have shown that an excess of heme in adipocyte stem cells and in the fat of obese mice is necessary in order for adiposity [11,33–37]. Therefore, increased heme levels in obese subjects, is a major source of ROS, contributing to lipid peroxidation and production of Ox-HDL and Ox-LDL. Additionally, hemoglobin influences LDL and HDL in obesity and diabetes. Hemoglobin increases the levels of proinflammatory HDL, in other words, increases the oxidation of HDL. We believe that HDL dysfunction is not the cause of adipogenesis, but it is the oxidation of the HDL itself [38].

Obesity is a growing epidemic in the United States as well as worldwide. Many of the cardiovascular complications associated with obesity are, in part, due to dysfunctional adipocytes and endothelial damage. Several clinical conditions such as diabetes mellitus and obesity, are characterized by both increased inflammation and oxidative stress, and are associated with increased risk of cardiovascular complications. An increase in Ox-HDL negatively correlated with adiponectin levels in morbidly obese subjects (unpublished data). Thus, HDL and Ox-HDL may prove of particular relevance, in the maintenance and regulation of cardiovascular health and as targets for the prevention of cardiovascular events.

In conclusion, this communication suggests that the novel finding that Ox-HDL and isoprostane act at the three points presented in figure 1, and that it appears that Ox-HDL enhances adipogenesis and/or the recruitment of stem cells in adipose tissue, and increases the adipogenic lineage and exacerbates obesity and the metabolic syndrome. In support of this conclusion, isoprostane, another oxidant found in the plasma of obese subjects increases adipogenesis and, with Ox-HDL, synergistically increases adipocyte stem cell proliferation, differentiation and hypertrophy. Thus Ox-HDL function, due to its adipogenic effect on adipocyte stem cells, should be re-evaluated to address the metabolic derangements associated with the metabolic syndrome.
